# Citizen involvement in COVID-19 contact tracing with digital tools: a qualitative study to explore citizens’ perspectives and needs

**DOI:** 10.1186/s12889-023-16664-x

**Published:** 2023-09-16

**Authors:** A. van der Meer, Y. B. Helms, R. Baron, R. Crutzen, A. Timen, M. E. E. Kretzschmar, M. L. Stein, N. Hamdiui

**Affiliations:** 1 National Coordination Centre for Communicable Disease Control, Centre for Infectious Disease Control, National Institute for Public Health and the Environment, Bilthoven, The Netherlands; 2 Julius Centre for Health Sciences and Primary Care, University Medical Centre Utrecht, Utrecht University, Utrecht, The Netherlands; 3 Department of Health Promotion, Care and Public Health Research Institute (CAPHRI), Maastricht University, Maastricht, The Netherlands; 4 Department of Primary and Community Care, Radboud Institute for Health Sciences, Radboud University Medical Center, Nijmegen, The Netherlands

**Keywords:** Contact tracing, COVID-19, Citizen involvement, Infectious diseases, Public health, Digital tools

## Abstract

**Background:**

Contact tracing (CT) is a key strategy when dealing with outbreaks of infectious diseases such as COVID-19. The scale of the COVID-19 pandemic has often left public health professionals (PHPs), who are responsible for the execution of CT, unable to keep up with the rapid and largescale spread of the virus. To enhance or support its execution, and potentially lower the workload for PHPs, citizens may be more actively involved in CT-tasks that are commonly executed by PHPs (referred to as ‘self-led CT’). There is limited insight into citizens’ perspectives on and needs for self-led CT for COVID-19. This study aims to explore the perspectives and needs of Dutch citizens on taking more responsibilities in the execution of CT for COVID-19, potentially through the use of digital tools.

**Methods:**

An exploratory qualitative study was performed, in which online semi-structured interviews were conducted. Questions were based on the Reasoned Action Approach and Health Belief Model. Interviews were audio-recorded and transcribed verbatim. A thematic analysis was conducted to identify citizens’ perspectives and needs to participate in self-led CT.

**Results:**

We conducted 27 interviews with Dutch citizens. Seven main themes were identified from the interviews: 1) ‘Citizens’ perspectives on self-led CT are influenced by prior experiences with regular CT’, 2) ‘Citizens’ felt responsibilities and the perceived responsibilities of the PHS in CT shape their perspectives on self-led CT’, 3) ‘Anticipated impacts of self-led CT on the CT-process’, 4) ‘Citizens’ attitude towards the application of self-led CT depends on their own perceived skills and the willingness and skills of others’, 5) ‘Shame and social stigma may hamper participation in self-led CT’, 6) ‘Concerns about privacy and data security: a barrier for self-led CT’, and 7) ‘Citizens’ perspectives and anticipated needs for the implementation and application of self-led CT in practice’.

**Conclusions:**

Most interviewees hold a positive attitude towards self-led CT and using digital tools for this purpose. However, their intention for self-led CT may depend on various factors, such as prior experiences with regular CT, and their perceived self-efficacy to participate. Perspectives and needs of citizens should be considered for the future implementation of self-led CT in practice.

**Supplementary Information:**

The online version contains supplementary material available at 10.1186/s12889-023-16664-x.

## Background

Contact tracing (CT) is a key strategy when dealing with outbreaks of infectious diseases that spread through physical contact between individuals [1]. In response to the COVID-19 pandemic, CT has been applied in many countries worldwide to identify and notify individuals who have had contact with individuals who are infected, to interrupt transmission chains of SARS-CoV-2, and, thereby, prevent further spread of the virus [2–5].

In the Netherlands, as well as in other countries, CT is carried out by public health services (referred to as “PHS”) [6]. Though the execution of CT in practice differs between countries, or even regions, it typically consists of three phases: contact identification, contact notification, and contact monitoring [7]. In the contact identification phase, an individual who has tested positive for, in this case, SARS-CoV-2 (further referred to as “index-case”) is contacted by a public health professional (PHP). The PHP provides information and advice (e.g., about isolation measures) and asks questions to identify individuals who were in close physical proximity to the index-case and who might, therefore, be at risk of infection with SARS-CoV-2 (further referred to as “contact persons”). Subsequently, in the contact notification phase, the PHP informs the listed contact persons, usually by phone, about 1) their exposure and infection risk, and 2) depending on the type of contact with the index-case, what measures should be taken to prevent further spread of the virus (e.g., quarantine). Finally, in the contact monitoring phase, the occurrence of any COVID-19-related symptoms among contact persons is monitored – ideally daily – for the (remaining) duration of the infectious period, which is generally about 10 days. Depending on the country and the guidelines/policies in place, different ways of monitoring are applied. Monitoring may be conducted by PHPs (active monitoring), through self-monitoring by the contact person and reporting to the PHS if symptoms occur (passive monitoring), or through self-reporting without reporting to PHS (self-monitoring). If a contact person does not experience COVID-19-related symptoms during the monitoring phase, the CT-process ends. If symptoms do appear, the contact person is requested to test for SARS-CoV-2. If tested positive, the contact person becomes an index-case, and the CT-process starts again with the contact identification phase [7, 8].

**Fig. 1:**
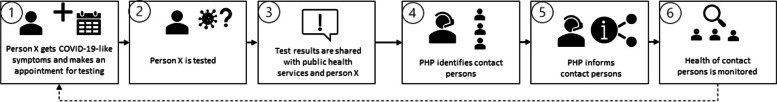
Schematic overview of the phases of the regular CT-process, including the contact identification (4),—notification (5), and—monitoring (6) phase. (CT = contact tracing; PHP = public health professional)

The execution of CT as described above (further referred to as ‘regular CT’) is a time and resource consuming activity that relies heavily on the workforce capacity of PHPs [2, 4, 5, 9]. See Fig. 1 for a schematic overview of the regular CT-process.

However, the scale of the COVID-19 pandemic – in terms of the number of confirmed cases – and the resulting workload for PHPs, has often left PHPs unable to keep up with the rapid and large-scale spread of the virus, in particular during ‘pandemic peaks’ [10]. To lower the burden on PHPs, index-cases and their contact persons may be given more responsibilities in the execution of regular CT, potentially through the use of digital tools, such as a mobile application or a website.

**Fig. 2:**
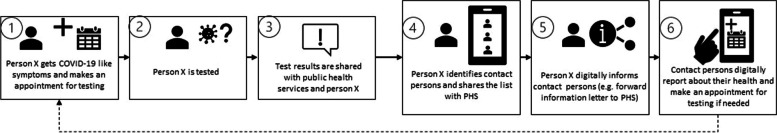
Schematic overview of the potential application of digital tools for self-led CT in the contact identification (4),—notification (5), and—monitoring (6) phase of the CT-process. (CT = contact tracing; PHS = public health services)

For instance, instead of a PHP calling index-cases to identify contact persons, index-cases may identify contact persons themselves and provide the PHP with a list of contact persons and their details. Instead of a PHP calling index-cases’ contact persons to inform them, index-cases may inform their own contact persons themselves by, for example, calling and/or forwarding an information letter provided by the PHS. Lastly, instead of a PHP calling contact persons to monitor symptoms, the contact persons may self-monitor and make an appointment for testing whenever symptoms occur. This approach, in which index-cases and/or contact persons assume more responsibilities for tasks that are commonly performed by PHPs and participate in CT more autonomously, potentially through the use of digital tools, is further referred to as ‘self-led CT’. See Fig. 2 for a schematic overview of self-led CT.

Studies investigating self-led CT such as described above are scarce, especially in the context of close-contact pathogens. Studies mainly focused on digital tools for monitoring index cases and/or contact persons’ symptoms and health status [11], or digital tools that automate the identification and notification of contact persons using GPS-location or Bluetooth signals that serve as an anonymous proxy for physical interactions between individuals [9, 12–14]. One study found that anonymous case-initiated contact notification may mitigate citizens’ concerns regarding privacy and reduce the stigma that can hamper contact notification [15]. Another study described the use of a contact diary web-application that citizens can use to manually record contact persons daily (to reduce recall bias), share information with the PHS, and notify contact persons, via e-mail [11]. Furthermore, various studies on digital partner notification in the context of CT for sexually transmitted infections (STIs) (i.e., patient-led contact notification) using e-mail, SMS and/or smartphone applications, found that it can reduce the workload for PHPs, accelerate the CT-process, and increase the number of sexual contacts reached through partner notification [16].

Although these studies indicate that digital tools for CT have the potential to enhance the CT-process, they typically do not focus on the principle of giving index-cases and/or contact persons more responsibilities and autonomy in CT (i.e., self-led CT). To date, it has not been (systematically) investigated if and how index-cases and their contact persons would be open to participating in self-led CT. Therefore, this study primarily aims to explore the perspectives and needs of Dutch citizens with regard to participating in self-led CT. Second, it aims to explore the potential use of digital tools for this purpose. In this study we will focus on CT for COVID-19. Our findings may also be relevant, however, for future outbreaks of other close-contact pathogens with similar (pandemic) characteristics as the COVID-19 pandemic, in terms of the large number of confirmed cases and the resulting workload for PHPs conducting CT.

## Methods

### Study design

In November 2021, an exploratory qualitative study was performed in the Netherlands. The reporting of this study was based on the Consolidated criteria for reporting qualitative research (COREQ) checklist [17]. Semi-structured interviews were conducted to investigate citizens’ perspectives and needs on participating in self-led CT. Due to the social distancing measures, because of the COVID-19 pandemic, which were in place in the Netherlands at that time, interviews were conducted online using two platforms called Cisco WebEx Meetings (V.41.10.2) and Whereby Meetings [18].

### Study population and sampling

We recruited Dutch citizens aged 16 years or older via purposive sampling, or more specifically ‘maximum variation sampling’, which strives for diversity in age, gender, educational level, migration background, and previous experience in CT [19]. The aim was to include a diverse sample, with regards to age, gender, educational level, migration background, and prior experience with CT for COVID-19 as an index-case and/or contact person. Interviewees were sampled via LinkedIn, a research panel called Happy Labs [20], the social network of the researchers involved in this study, and previous research participants who had indicated willingness to participate in similar research projects. Potential interviewees were invited and informed about the objectives and methodology of the study via a digital information letter. Individuals who were willing to participate were explicitly asked to agree to the terms of the study through a digital informed consent form. To gain more context for the views and perspectives provided, interviewees were then asked to complete several questions regarding socio-demographic characteristics such as age, gender, educational level, (parental) country of birth and experience with CT for COVID-19 (i.e., were they involved in CT as an index-case and/or contact person, and how long ago was this?). We categorized educational level as low, medium, and high. “Low” is defined in the Netherlands as primary education, preparatory secondary vocational education, senior secondary general education, pre-university education, or senior secondary vocational education; “medium” is defined as senior secondary general education, pre-university education, or senior secondary vocational school; and “high” is defined as higher professional education or academic higher education [21]. Besides individuals who were born in the Netherlands, those who were born abroad (also called first generation individuals with a migration background), and those born in the Netherlands with at least one parent who was born abroad (also called second generation individuals with a migration background) were included. All individuals to whom we sent the digital informed consent form and questionnaire participated in this study.

### Data collection

Online interviews were conducted by two researchers (AM and NH) and lasted approximately one hour.

The interview guide was based on the Reasoned Action Approach (RAA) [22] and the Health Belief Model (HBM) [23], to explore the perspectives and needs of Dutch citizens with regard to participating in self-led CT. The RAA is the latest version based on the work by Fishbein and Ajzen (2011). ‘*Intention’* is central in RAA and captures ‘how hard an individual is willing to try, how much effort he/she would exert to perform a certain behaviour’ [23–25]. In general, the literature provides strong evidence for the model’s effectiveness in the predictions of intentions and is considered to be broadly supportive in helping to understand and predict health behaviours [26]. This theory is, therefore, also used to develop a conceptual framework for this study. The HBM aims to predict whether and why people will take action to prevent, to screen for, or to control illness conditions [23], and is generally targeting individuals at “health-risk”, that are already sick, or used to develop interventions that improve health-related behaviour [27]. We believe self-led CT aligns with the aims of both models. However, since there is overlap between the HBM and RAA, we mainly used the RAA. Additionally, since the use of digital tools for self-led CT was our secondary focus, we chose not to include technology-focused models for this study (e.g., the Unified Theory of Acceptance and Use of Technology, UTAUT).

Following the RAA and HBM, we assumed that a citizen’s intention to participate in self-led CT is influenced by 1) background/modifying factors (i.e., individual factors such as perceived COVID-19 risk, and general attitude towards CT for COVID-19 and digital technologies), socio-demographic factors such as age, gender, migration background, and educational level, and information factors such as knowledge of—or experience with CT for COVID-19, 2) citizens’ attitude towards self-led CT, 3) citizens’ perceived norm regarding the involvement in self-led CT, and 4) citizens’ perceived behavioral control. An overview of the definitions of the conceptual framework can be found in Table 1 below.

**Table 1:** Definitions of the conceptual framework

Construct	Definition and operationalization	Interview guide question examples
Intention to participate in self-led CT	Intention represents a person’s motivation and, in this study, refers to an individual’s plan or decision to exert effort in self-led CT [28]. Intention is influenced by background/modifying factors, as well as their attitude, perceived norm, and perceived behavioural control	See other constructs
Background/modifying factors	Background or ‘modifying’ factors may influence perceptions and, indirectly, health-related behaviour [22, 23]. For this study, we focused on 1) individual factors such as perceived COVID-19 risk (including perceived severity and—susceptibility) and general attitude towards CT and digital tools for CT, 2) socio-demographic factors such as age, gender, ethnicity, and educational level, and 3) information factors such as knowledge of or experience with CT	*Are you familiar with the term ‘contact tracing’?* *What is your opinion on contact tracing?*
Attitude towards self-led CT	Following the RAA, attitude is determined by an individuals’ behavioural beliefs [22], which may include beliefs about (anticipated) positive or negative outcomes of performing the behaviour, and beliefs about how the behaviour is/will be experienced. This was explored by discussing the citizens’ perceived benefits (e.g., belief in efficacy of self-led CT) and perceived barriers (e.g., belief about tangible/psychological costs of self-led CT)	*How would you feel about this* [contact identification in self-led CT]*?* *What do you think would be (dis)advantages of doing this* [contact identification in self-led CT]*?*
Perceived norm	Perceived norm is determined by individuals’ ‘normative beliefs’. If more important others are believed to approve than disapprove a certain behaviour (injunctive norm), and if the majority of these persons perform the behaviour (descriptive norm), individuals are likely to perceive social pressure to engage in that certain behaviour [22]	This was not explicitly included in the interview guide but may come forward as a perceived barrier or facilitator when individuals’ attitude towards self- led CT is explored (as literature suggests) [29–33]
Perceived behavioural control	Perceived behavioural control refers to individuals’ beliefs about the degree to which they are capable, or have control/autonomy over performing a certain behaviour [34, 35]. Individuals who believe they have neither the resources nor opportunities to perform a certain behaviour are unlikely to have high intentions to perform the behaviour, even if they hold a favourable attitude towards the behaviour, or if perceive favourable subjective norms for the behaviour [36] In this study, perceived behavioural control was operationalized as factors that make it easy or difficult for citizens to participate in self-led CT	*What would make it easier for you to do this* [contact identification in self-led CT]*? And what would make it more difficult?* *What would you need to identify your contacts as accurately as possible* [in self-led CT]*?*

*CT* Contact tracing, *RAA* Reasoned Action Approach

Interviews consisted of four parts. First, exploratory questions regarding citizens’ experiences with – and knowledge of CT for COVID-19 were asked. Second, regular CT was briefly explained in a step-by-step fashion using the first part of a PowerPoint presentation designed for this purpose. Through this presentation, we aimed to ensure that all interviewees had an understanding of regular CT at the start of the interview. Third, the interviewees’ perceptions on the possibilities to take more actions, control, and responsibilities in the execution of CT for COVID-19 (i.e., self-led CT) were discussed. For each separate CT-phase (i.e., contact identification, -notification, and -monitoring), interviewees were asked whether and how they would consider taking more actions, control, and responsibilities in CT and why or why not. The second part of the PowerPoint presentation showing each CT-phase was used to visually support the interviewee throughout the interview. Fourth, the interviewees’ perceptions on the possibilities to take more actions/control and responsibilities in self-led CT with digital tools were discussed. Again, for each separate CT-phase, interviewees were asked whether, how, and why or why they would not consider using digital tools in practice for self-led CT. The third part of the PowerPoint presentation showing the potential application of digital tools in each CT-phase was used to visually support the interviewee throughout this part of the interview.

The interview guide and the materials used during the interviews were pilot tested online with two interviewees. As a result of the pilot interviews, minor adjustments to the phrasing and order of the questions were made. The interview guide can be found in Additional file 1.

Each interviewee was given a token of appreciation in the form of a gift voucher worth 10 euros for their participation in the study.

### Data analysis

The interviews were audio-recorded using the recording tool available in the beforementioned meeting platforms, and transcribed verbatim. Interviews were analyzed inductively using the qualitative software program MAXQDA v.20.0.7 (Berlin: VERBI GmbH). The analysis focused on citizens’ perspectives on participating in self-led CT, their expected needs in practice, their beliefs about self-led CT and their anticipated barriers and facilitators to participate in self-led CT. The analysis was based on the principles of thematic analysis, which allows the use of both pre-defined themes obtained from theory, as well as new inductive themes that arise from the interview data [37]. First, transcripts were coded by labelling relevant fragments of text (i.e., open coding). All interviews were open coded by AM, of which ten in total were double coded by NH and YBH to reduce the subjective interpretation of data. Thereafter, themes and subthemes were identified through systemic comparison of the coded text (i.e., axial and selective coding). Interpretation of the themes and subthemes were discussed among the researchers until consensus was reached.

## Results

### Sample characteristics

We conducted 27 interviews with Dutch citizens (see Table 2). No new (sub-)themes were identified after 11 interviews. Sixteen more interviews were conducted nevertheless, since these had already been planned, and we intended to maximize the sample’s diversity. Although we looked for patterns between interviewees’ socio-demographic characteristics and their perspectives and needs regarding (digital) self-led CT, we did not find any.

**Table 2:** Interviewees’ socio-demographic characteristics

Characteristics	Interviewees (*n* = 27) n (%)
Age in years	
• 16 – 35	9 (33)
• 36 – 55	15 (56)
• 56 +	3 (11)
Gender	
• Male	6 (22)
• Female	21 78)
Educational level	
• Low	1 (4)
• Medium	10 (37)
• High	16 (59)
Migration background	
• Yes, 1^st^ generation	5 (19)
• Yes, 2^nd^ generation	11 (41)
• No	11 (41)
Experience with CT as an index-case	
• Yes	15 (56)
- Contacted by PHS for CT	
◦ Yes	12 (80)
◦ No	3 (20)
• No	12 (44)
Experience with CT as a contact person	
• Yes	15 (56)
- Contacted by PHS for CT	
◦ Yes	6 (40)
◦ No	9 (60)
• No	12 (44)
No experience with CT as an index-case, nor as a contact person	7 (26)

*CT* Contact tracing, *PHS* public health services

### Themes and subthemes

Seven main themes related to the perspectives and needs of Dutch citizens on self-led CT were identified from the interviews.

Three themes included subthemes. An overview of all themes and subthemes can be found in Table 3 below.

**Table 3:** Overview of themes and subthemes

Themes	Subthemes
1. Citizens’ perspectives on self-led CT are influenced by prior experiences with regular CT	N/A
2. Citizens’ felt responsibilities and the perceived responsibilities of the PHS in CT shape their perspectives on self-led CT	N/A
3. Anticipated impacts of self-led CT on the CT-process	1. Efficiency of CT may be increased with self-led CT 2. Diverging perspectives on the accuracy of contact identification with self-led CT 3. Positive perspectives on self-monitoring
4. Citizens’ attitude towards the application of self-led CT depends on their own perceived skills and the willingness and skills of others	1. Trust in one’s own ability to participate in self-led CT’ 2. Negative expectations of others’ ability and willingness to participate in self-led CT
5. Shame and social stigma may hamper participation in self-led CT	N/A
6. Concerns about privacy and data security: a barrier for self-led CT	N/A
7. Citizens’ perspectives and anticipated needs for the implementation and application of self-led CT in practice	1. Citizens perceive self-led CT as complementary to regular CT 2. Preference to tailor CT-approach per individual contact person 3. Citizens’ needs for the use of self-led CT in practice

*CT* Contact tracing, *N/A* Not applicable

#### Theme 1: Citizens’ perspectives on self-led CT are influenced by prior experiences with regular CT

Almost all interviewees were familiar with the concept “CT for COVID-19”. They could (broadly) explain what it entails and understood the goal of CT (i.e., prevent further spread of SARS-CoV-2 and gain insight in transmission routes). However, several interviewees, mainly those with no personal experience of CT for COVID-19, were not familiar with the contact monitoring phase and saw CT only as a process in which contact persons are identified and notified. In general, all interviewees were open to participating in self-led CT, yet the reasons and motivation to do so varied between interviewees and appeared to have been influenced by their previous positive and/or negative experiences with regular CT for COVID-19.

Most interviewees who participated in CT reported ‘positive CT-experiences’. They frequently described these experiences as being informative and comprehensible conversation with a PHP, held during an appropriate time of the day and of limited duration, but still with sufficient opportunity to ask questions. Interviewees with positive CT-experience(s) generally had positive attitudes towards self-led CT, because they felt that it may improve the CT process in terms of efficiency (i.e., saves time and work for PHS) and effectivity (i.e., contact persons are informed more quickly). For the interviewees, participation in self-led CT appeared to be conditional on having the opportunity to contact a PHP for support if needed or preferred. This would give them the opportunity to ask questions, to share concerns, to check the completeness of their list of contact persons, or to let the PHP notify ‘difficult contact persons’ (e.g., individuals who are more ‘distant’ from the index-case in terms of their relationship).

*“You should give someone the opportunity to ask a question directly to a PHP. Everyone deserves to have something to fall back on if they are experiencing difficulties* [during self-led CT].” – Male, early 30s (interview 25)

A few interviewees had a relatively negative CT-experience. They felt that the phone call with the PHP took too much time, that the information shared by the PHP was too extensive and too complex, or that the communication with the PHP came across as impersonal. Nevertheless, similarly to interviewees with a positive CT-experience, most interviewees with a negative CT-experience seemed to be willing to participate in self-led CT. However, in contrast to individuals with a positive CT-experience, their main motivation appeared to be that it would provide them with the opportunity to have more control over the CT-process and it would make them less dependent on the PHP.

*“I would absolutely choose a mobile application over a phone call. Calling takes time and I would rather spend my time on my children, work or things that are more important than on a PHP telling me about COVID-19. These calls take way too long.”* – Female, mid 30s (interview 4)

Notably, all interviewees who were previously involved in CT as an index-case reported that they had already begun with identifying and notifying (some of) their contact persons on their own initiative—prior to the phone call with a PHP. Some respondents felt that the phone call with the PHP was redundant, as they had already notified their contact persons about their risk of infection and the measures that had to be taken.

#### Theme 2: Citizens’ felt responsibilities and the perceived responsibilities of the PHS in CT shape their perspectives on self-led CT

Interviewees’ perspectives on responsibilities in the different phases of the regular CT-process appeared to influence their perspectives on self-led CT in practice. In general, participation in CT was considered a shared responsibility between citizens and the PHS, in the sense that interviewees see it as a collaboration between the two. Often interviewees felt that it was ‘their duty’ as a citizen (but also that of other citizens) to participate in regular CT and to contribute to controlling the spread of COVID-19.

The identification and notification of contact persons was mainly seen by the interviewees as an individual responsibility of citizens, rather than the PHS’ responsibility. Interviewees felt that their contact persons have the right to know about their possible risk of infection. They considered it their ‘obligation’ to be open about it and to actively participate in the identification and notification of their contact persons, and thus had a positive attitude towards their participation in self-led CT. This also applied to the contact monitoring phase: it was generally felt by interviewees that the PHS should not have to ‘monitor’ every action of each contact person, because monitoring one’s own symptoms was mainly seen as one’s ‘own responsibility’.

“*I think people should be responsible for the monitoring of their symptoms themselves, and if they develop symptoms that they get a test immediately.*” – Female, late 40s (interview 1)

In contrast, a few interviewees felt that the notification of contact persons would be too much responsibility for the index-case.

*“Well, I don’t mind it* [self-contact notification]*, but I feel like the responsibility is put on you* [as a citizen]*. I might be able to handle it, but someone else might not. For example, the information may be shared in the wrong way. I think that is something serious.”* – Female, mid 40s (interview 23)

It was generally felt that the PHS should provide support and guidance to index-cases and contact persons throughout the CT-process, if needed or preferred. A few interviewees also reasoned that their ‘relative low sense of responsibility’ to participate in self-led CT would increase if the infection rate was high and the PHS did not have sufficient time to fully facilitate CT.

#### Theme 3: Anticipated impacts of self-led CT on the CT-process

Interviewees anticipated that giving index-cases and contact persons more responsibilities and a more active role in CT would impact the CT-process in different ways. Their expectations in this regard appeared to be of influence in their perspectives on self-led CT.

#### Subtheme 1: Efficiency of CT may be increased with self-led CT

In general, interviewees indicated that if the CT-process was executed more efficiently through a self-led approach, they would be more willing to participate. For example, if the index-case was responsible for the identification and notification of their contact persons (instead of the PHP), this could result in a faster provision of the contact persons’ information to the PHS, and contact persons could be reached and be aware of what measures to take more quickly. Additionally, it was felt that self-led CT would save PHPs time and work not having to reach out to each index-case and their contact persons. Interviewees also believed that it may save time for index-cases not having to wait for the PHP to contact them and spend time on the phone.

*“You will be much quicker if you inform your contacts yourself. Otherwise, it would take two more days for you to wait for the PHS.”* – Female, late 40s (interview 1

#### Subtheme 2: Diverging perspectives on the accuracy of contact identification with self-led CT

Interviewees generally believed that the accuracy of contact identification would increase with self-led CT. If the index-case is responsible for the identification of contact persons, they would have the opportunity to write down (or type in – when using digital tools) contact information and check the list for mistakes. Compared to regular CT, interviewees felt that in self-led CT, the index-case also had more time to think about one’s contact persons and to gather all the required contact information. This would result in a more accurate delivery of information about the contact persons to the PHS.

By contrast, a few interviewees felt that the accuracy of the contact information may possibly decrease with self-led CT. They had doubts about whether the identification of contact persons should be the individual responsibility of index-cases. For example, they anticipated that the index-case may forget or misclassify contact persons, may deliberately not report certain contact persons, or may not be willing to identify their contact persons at all. Interviewees stated that this was different from regular CT, where the PHP could use professional questioning skills to avoid inaccurate information about one’s contact persons.

“*There could be people that will forget about the persons they have seen. So, the chances of contact persons ‘slipping away’ may be bigger without the help of a PHP*.” – Female, late 20s (interview 10)

#### Subtheme 3: Positive perspectives on self-monitoring

Regarding the contact monitoring phase, interviewees felt that self-monitoring may generally improve the awareness of symptoms and possibly lead to contact persons getting tested more quickly. Also, writing down symptoms, or filling symptoms out in an app or website provides an opportunity for contact persons to gain insight into the possible progression of their symptoms.

“*I can monitor my own symptoms. If I think they are getting worse, I will act on that. I do not need anyone else to remind me to check on myself*…” – Female, late 30s (interview 3)

In contrast to positive perspectives on self-monitoring, two interviewees anticipated that contact monitoring may also be executed too meticulously, leading to ‘over-awareness’. To avoid this over-awareness, or overreporting, interviewees stated that it would be important to inform individuals well about the types of symptoms to self-monitor and about the steps to take if certain symptoms appeared.

#### Theme 4: Citizens’ attitude towards the application of self-led CT depends on their own perceived skills and the willingness and skills of others

Citizens’ own perceived skills and the perceived willingness and skills of others, appeared to influence interviewees’ attitudes towards self-led CT. This was related to trust in their own ability to participate in self-led CT, and their expectations of others’ competence and compliance.

#### Subtheme 1: Trust in one’s own ability to participate in self-led CT

Most interviewees regarded their own ability to identify and inform their contact persons more highly than that of a PHP. However, a few interviewees were doubtful about their ability to provide contact persons with the correct information. They felt that it would be challenging to know what information to share with what type of contact person and what measures might be needed, especially since these were constantly changing during the pandemic.

“*The rules* [COVID-19 measures] *are constantly changing, so I am afraid that I would give out the wrong information.*” – Female, early 40s (interview 2)

Furthermore, interviewees felt that their participation in self-led CT would depend on the number of contact persons. The identification and notification of many contact persons would cost the index-case too much effort and time, especially if they were experiencing severe COVID-19 symptoms. Interviewees also felt that their ability to inform contact persons would depend on the reachability of the contact persons. It was anticipated that especially elderly contact persons (with no access to a smartphone or the Internet), contact persons with limited digital skills, or a low affinity with apps, contact persons with a migration background (e.g., language barrier), contact persons who are sick or dependent, and contact persons with a negative attitude towards CT measures and/or the PHS may be difficult to reach and to be informed by the index-case. In contrast, a few interviewees mentioned that contact persons with a migration background who have been in contact with an index-case who is a friend or family member with a related background and who speaks the same language, are best informed by them personally instead of by a PHP.

In addition, not all interviewees trusted their own ability to make use of digital tools for the notification of their contact persons. Especially interviewees above the age of 55 years preferred having contact with their contact persons via a phone call, as this was considered more ‘personal’ and ‘human’.

*“I prefer personal contact* [over digital means]*. We are already living in a very impersonal time where everything goes *via* phone and apps.”* – Female, mid 70s (interview 11)

#### Subtheme 2: Negative expectations of others’ ability and willingness to participate in self-led CT

Interviewees were doubtful about the ability and willingness of others to participate in self-led CT. For example, they felt that individuals may not be motivated, or able to take the responsibility to 1) identify their contact persons in an accurate and complete manner, 2) inform their contact person(s) in a correct manner, or 3) regularly monitor symptoms accurately and get tested if needed. These doubts did not seem to influence for interviewees’ own participation in self-led CT, but participants mentioned it as having influenced their attitude towards the applicability of self-led CT for the general public.

*“I can imagine that others may think ‘I am not going to do that* [contact identification and – notification]*’. They may not dare to or simply do not feel like it and are a bit laxer. So, I am afraid that not everyone will be reached.”* – Female, late 40s (interview 14)

#### Theme 5: Shame and social stigma may hamper participation in self-led CT

Interviewees’ intention to participate in self-led CT may depend on shame regarding their COVID-19-infection status and their perceived social stigma associated with a COVID-19 infection.

Several interviewees were afraid of having infected others (who may become seriously ill) or ‘putting others in quarantine’. They reported preferring not to draw too much attention to their COVID-19-infection, because they expected that they would feel shame and guilt for being the cause of others’ risk of infection. This was a strong anticipated barrier for index-cases to reach out to their contact persons and notify these individuals.

*“What if this person has fragile or poor health? Then I would think, how am I going to tell him/her this* [possible risk of infection]*?”* – Female, early 40s (interview 2)

Conversely, for several interviewees, the ‘fear of having infected others’ had a facilitating role on their anticipated participation in contact notification, since they were willing to inform their contact persons as quickly as possible to prevent further spread of the virus and more individuals getting infected.

*“Imagine if you did not do it* [contact notification] *for whatever reason, and afterwards you find out that he or she gets infected* [by COVID-19]*. I would be very ashamed of myself, and I would feel very guilty.”* – Female, late 30s (interview 7)

Furthermore, interviewees noted that notification conducted by the index-case would allow contact persons to express their emotions to the index-case. Such emotional expressions could on the one hand be positive, leading to appreciation of being notified by contact persons. On the other hand, emotional expressions could be negative, as contact persons may not be pleased to hear about their risk of infection and the possible required measures to take. Some interviewees feared that contact persons may blame the index-case for having complied with the COVID-19 measures and that they may be seen as a ‘spreader’. Some even feared being ‘socially isolated’, due to their infection. Interviewees indicated that these negative emotional expressions had an impact on their willingness to notify their contact persons themselves.

*“The reaction* [of the contact person(s)] *may be an issue, in a way that their reaction could be very uncomfortable for me. Maybe I would even not want to do it* [contact notification]*.”* – Female, late 30s (interview 3)

*“It* [information about a possible risk of COVID-19 infection] *is not a fun thing to tell someone. People can blame you for infecting them. What if they end up at the ICU and die? People may say ‘she* [interviewee] *is to blame for this’.”* – Female, early 40s (interview 26)

#### Theme 6: Concerns about the privacy and data security: a barrier for self-led CT

Interviewees stated that concerns about personal privacy, the privacy of others, and the security of CT-data, influenced their willingness to participate in both regular CT and self-led CT. Whilst most interviewees indicated that they would have no issues sharing their own (personal) information with the PHS, they anticipated more difficulties in sharing information of others with the PHS. For example, almost all interviewees described feeling uncomfortable about sharing personal data of their contact persons without their permission to do so. Most of them were, however, willing to share these data after obtaining permission from their contact persons.

*“It does not feel right to share contact details with organizations without having the permission to do so.* […] *So, I would feel uncomfortable to share names with the PHS, because I have not asked them* [contact persons] *if they would be OK with it. I would personally not like that either* [if someone else would share the interviewee’s contact details without permission]*.”* – Male, mid 30s (interview 9)

In addition, sharing details with the PHS over the phone was less of a barrier to some interviewees, compared to sharing data through digital tools. These interviewees appeared to have a positive attitude towards the identification of contact persons and sharing their contact details with the PHS via a phone call, but not via digital tools. For example, interviewees spoke of their mistrust and/or apprehension towards digital tools for self-led CT and described a fear of being traced or controlled, not being able to get rid of a (mobile or web) application, or a fear of the tools being unsafe (i.e., data may leak). A few interviewees also mentioned having too many apps on their smartphone, which hampered their willingness to make use of more apps and/or digital tools for self-led CT.

*“No, I don’t think I would do that* [make use of a digital tool for CT], *due to privacy. I don’t know if I would just put contact details of my friends, acquaintances, family in an app.”* – Female, late 30s (interview 5)

A few interviewees noted that the whole ‘privacy problem’ could be solved if index-cases could notify their contact persons themselves, without having to share their contact persons’ information with the PHS.

#### Theme 7: Citizens’ perspectives and anticipated needs for the implementation and application of self-led CT in practice

In general, interviewees had a positive attitude regarding the implementation of self-led CT in practice. They perceived self-led CT as being complementary to regular CT, but they preferred the CT-approach to be tailored to the individual. Additionally, interviewees spoke of several needs for the use of self-led CT in practice.

#### Subtheme 1: Citizens perceive self-led CT as complementary to regular CT

Interviewees generally stated that the entire regular CT-process cannot be replaced by self-led CT, and CT may only be executed partly by index-cases and contact persons. Interviewees worried that leaving the full responsibility to index-cases and contact persons, as well as complete digitalization, would exclude certain groups in society (e.g., the elderly, people with limited digital skills, or people who are sick or dependent). As it also involves less direct contact with a PHP during CT, it would result in index-cases having fewer opportunities to ask questions, and to share their worries or needs. Therefore, it was felt that regular CT should continue to exist, but that individuals should have the option to choose whether, and to what degree they would want to perform parts of the CT-process themselves, and whether they would make use of digital CT-tools for this purpose.

“…*I think especially for the persons that find it* [self-led CT] *difficult to do execute steps within CT themselves, also via an application. And this does not only account for elderly. I think it is important to reach as many people as possible,* [in CT] *so providing both options* [regular CT and self-led CT] *would be good.*” – Female, mid 60s (interview 21)

If they chose to participate in self-led CT, interviewees regularly stated that it should still be easy for index-cases and their contact persons to consult a PHP if needed or preferred (e.g., via a web-link or telephone number). They also stated that there should be a manner to provide the PHS information about which contact persons have been notified, so that the PHS has insight into the progression of the CT-process.

#### Subtheme 2: Preference to tailor CT-approach per individual contact person

We identified three pillars related to the individual contact person, which shaped interviewees’ approach for self-led CT: the anticipated attitude of contact persons towards CT-policies and the PHS, the personal relationship to the contact person, and the contact person’s individual characteristics.

First, interviewees’ expectations about the attitude of their contact persons towards both CT policies and the PHS appeared to affect their willingness to participate in self-led CT. If contact persons were expected to 1) not take the PHS or the COVID-19 measures seriously, 2) not fully support the COVID-19-measures, or 3) not see the added value of CT for COVID-19, they imagined two possible scenarios. On the one hand, interviewees anticipated that such contact persons may only listen to them, instead of an ‘unknown’ PHP, and would take the information more seriously when personally informed by the index-case. This could especially be the case for contact persons who were expected to perceive notification by the PHS as ‘intrusive’. On the other hand, interviewees would rather have a PHP inform these ‘difficult contact persons’, as they anticipated adverse reactions or difficult questions from their contact persons. Additionally, they perceived the PHS to have authority as an ‘official institute’ and to come across as more reliable than the index-case for these contact persons.

Second, the personal relationship with the contact person seemed to influence interviewees’ preferred approach for self-led CT. They felt that ‘close’ contact persons (i.e., individuals they ‘care about’) should be informed by the index-case, as personal contact may be more reassuring and less overwhelming for these contact persons. It may be easier for close contacts to talk and share their feelings and thoughts with a person they know and trust, instead of a PHP they do not personally know.

*“Well, it* [informing close contact persons] *is a bit more personal. You have greater trust in your friends* [compared to a PHP]*.”* – Female, mid 30s (interview 4)

On the contrary, most interviewees reported that they would prefer the PHS to notify their more ‘distant’ contact persons (e.g., colleagues and friends of friends). They anticipated that it would be more difficult to reach these contact persons due to practical and social reasons, including having no access to their contact details, not wanting to disturb them and fear of how they might react.

Third, interviewees felt that their choice to participate in self-led CT depended on their contact persons’ individual characteristics. These characteristics mainly included language skills, health status, general (health) knowledge, digital skills, affinity with digital tools, and access to digital technologies (such as a smartphone and the Internet). To illustrate, interviewees believed that contact persons who are unlikely to understand the information about the possible exposure and the required CT-measures, should be notified by the PHS, as they are able to provide them with the correct information and allow contact persons to ask questions. Contact persons with a migration background were an exception to this. As mentioned before, contact persons with a migration background who had been in contact with an index-case who was a friend or family member with a related background and who spoke the same language, were best informed by them personally instead of by a PHP.

Regarding the use of digital tools in self-led CT, interviewees believed that contact persons who may be difficult to reach through digital means should preferably be called, instead of being notified via digital means (e.g., a digital information letter, text message, message via WhatsApp, or email).

#### Subtheme 3: Citizens’ needs for the use of self-led CT in practice

Anticipated needs for citizens for self-led CT in practice were regularly based on previous experiences with CT for COVID-19. To illustrate, interviewees who experienced confusion about CT-guidelines or who received too much or inconsistent information from the PHS, expressed a need for clear and brief instructions and information regarding CT. Interviewees believed it was appropriate to design digital CT-tools in the form of a web-based or mobile-phone app that took privacy and safety of data into account and explicitly explained this to the user. More specifically, several interviewees stated that their trust in the safety of their data would increase if the PHS could explicitly guaranty the safety of their data used, for instance by providing them with a ‘data safety certification’.

“*For example, an indication of the extent to which your data is safe. If such a thing exists, something like a quality mark for apps. That you are able to weigh up the safety of the app: is this safe enough for me or not?*” – Female, mid 30s (interview 19)

Interviewees also described the importance of the design being user-friendly, meaning that the app should be easily installed and set-up, and texts or questionnaires within the app should be short and easily understandable, for example, supported by visual information. Interviewees anticipated that the needs regarding the implementation of digital CT-tools would differ between CT-phases. First, for the contact identification and –notification phase, there is a need for the following: 1) practical support in the identification and notification of contact persons for index-cases, 2) information about the added value of digital identification and notification of contact persons, 3) the possibility to adjust the list of contact persons and personal information as well as sharing it again with the PHS, 4) support for index-cases in choosing the correct information letters to forward to their contact persons, and 5) tailored information letters (i.e., adjusted to the category a contact person falls into) that are both easily accessible and understandable for contact persons. Second, for the contact monitoring phase, interviewees expressed a need for the following: 1) a reminder to monitor symptoms regularly (every couple of days, not daily), 2) the option to fill out COVID-19-related symptoms using a checkbox, and if needed, detailing them in a textbox, and, lastly, 3) clear information about what to do when COVID-19-related symptoms occur.

## Discussion

### Main findings

This pioneering study investigated the perspectives and needs of citizens regarding opportunities for index cases and contact persons to become more involved in the CT-process. Our results showed that most interviewees held a positive attitude towards self-led CT and using digital tools for this. However, their willingness to make use of self-led CT appeared to depend on their prior experiences with regular CT, their felt responsibilities and perceived responsibilities of the PHS in CT, their anticipated impacts of self-led CT on the CT-process, their own perceived skills and the willingness and skills of others, the extent to which they felt shame and social stigma, and their concerns about privacy and data security. Additionally, interviewees anticipated that self-led CT may be less applicable in situations with many contact persons, or in situations that involve individuals who are elderly, illiterate, or unable to communicate in Dutch. Furthermore, interviewees felt that self-led CT could not replace the entire CT-process, and CT may only be executed partly by index-cases and contact persons. They saw self-led CT as complementary to regular CT, but that the CT-approach should be tailored to the individual. When using digital CT-tools, interviewees felt that these may only be applicable in situations that involve individuals who have access to – and are familiar and experienced with – such tools (e.g., a smartphone and the Internet). The privacy and safety of their data used for CT should be explicitly guaranteed in this regard.

Previous research among Dutch PHPs [38, 39] investigated if, why, and how index-cases and contact persons could more actively and autonomously support the execution of CT for COVID-19 or other infectious diseases through digital CT-tools in a similar fashion as the current study. Their findings demonstrated that PHPs seemed to have an overall positive attitude towards the application of such tools, in all stages of the CT-process. They expressed, however, a fear of losing oversight and control over the CT-process when giving citizens more autonomy and responsibilities in CT and preferred to receive as much data as needed for CT from the index-cases and contact persons. When comparing these findings with our findings, we primarily see that both citizens and PHPs had a positive attitude towards giving more responsibilities to index-cases and contact persons in CT. Additionally, citizens expressed a need for the possibility to contact a PHP if needed or preferred. This could, to a certain extent, help counteract the fear of the PHPs in losing oversight and control over the CT-process. However, findings from the current study also showed that citizens may not always be willing to share (all) data about themselves or their contacts persons with the PHS, because of privacy and data safety reasons. This may potentially hinder PHPs in the receival of as much data as possible for CT.

Further insights specifically related to citizens’ intention to make use of digital tools in self-led CT are scarce. Previous studies mainly focused on digital tools for monitoring index cases and/or contact persons’ symptoms and health status [11], or ‘automatic digital CT-tools’, aiming to automate the identification and notification of contact persons who may be at risk of an infection through an automatically recorded GPS-location or Bluetooth signals that serve as a proxy for physical interactions between individuals [9, 12–14]. These studies show similar determinants of the intention to accept and adopt digital tools for CT, such as privacy and security concerns [31, 40], feelings of shame and stigma [41], trust in technologies for CT [42–44], social responsibility [29, 31], performance expectancy, perceived benefits [29], and understanding [40, 44]. More specifically, it was found that users need to have trust in their data being stored safely and that such tools are only used for the control of COVID-19 [43]. Besides this, they need to have experience in -, access to -, and competence in using digital tools (i.e., “digital literacy”), to understand and believe in the potential benefits of such tools, and to be motivated to contribute to the greater good (i.e., the public health) [31, 33]. More extensive research on digital CT-tools has been conducted in the context of sexually transmitted infections (STIs). For example, various studies on digital partner notification services (i.e., self-led contact notification in the context of STIs) using e-mail, SMS and/or smartphone applications, found that such services can reduce the workload for PHPs, accelerate the CT-process, and increase the number of sexual contacts reached through partner notification [16, 45–49].

To a certain extent, the findings described above are comparable to the current study and may be useful to consider when it comes to implementing digital tools for self-led CT in practice. However, there are distinctions that should be kept in mind. Compared to digital tools in self-led CT, automated tools do not fully require citizens’ active engagement, and the identification and notification of contact persons is automatic and anonymous [50, 51]. In self-led CT index-cases are responsible for the identification and notification of their contact persons themselves, in a non-anonymous way, which requires more effort and time for the index-case. Findings of the current study show that this distinction may result in additional determinants of the intention to participate in self-led CT, such as the extent of trust in one’s own knowledge and skills to participate, the closeness of one’s relationship to the contact person, or having enough energy and time to participate in self-led CT. Furthermore, although studies in the context of STIs yielded similar findings, CT for STIs and CT for close-contact pathogens are different in terms of exposure-risk guidelines and contact definitions, the numbers of at-risk contacts, and the extent of shame and stigma that can influence the CT-process. This should be kept in mind when comparing these findings to the findings of the current study, during further work on self-led CT and the implementation of digital tools for this purpose.

### Strengths and limitations

An important strength of this study is that we conducted interviews with a diverse sample of citizens. Since we recruited interviewees through various channels, we also managed to reach individuals with a first- and second-generation migration background who are often under-represented in studies. Another strength is that we conducted interviews during the COVID-19 pandemic, which provided unique insights into the perspectives and needs of citizens when it comes to participating in self-led CT. Despite the fact that the COVID-19 pandemic is now behind us, we believe that our findings may also be relevant for future outbreaks of other close-contact pathogens that have similar characteristics as the COVID-19 pandemic, since it is close to certain that more pandemics will arise in the future [52]. Additionally, the interview guide used in this study was mainly based on the Reasoned Action Approach, with additions from the Health Belief Model. Even though we performed an inductive thematic analysis, we did not find themes that we could not relate back to the main constructs of these theories (see Table 4). We, therefore, believe that there is a good conceptual match between the study matter and questions and the theories that we started with. The interview guide and additional materials used to explain self-led CT to interviewees were pilot tested among two citizens. This improved the understandability of the interview guide and the additional materials.

**Table 4:** Comparison between themes found in this study and main constructs of RAA and/or HBM

Themes found in this study	Conceptual relationship with main constructs from RAA and/or HBM (operationalized in this study as)
1. Citizens’ perspectives on self-led CT are influenced by prior experiences with regular CT	Background factors (Background/modifying factors)
2. Citizens’ felt responsibilities and the perceived responsibilities of the PHS in CT shape their perspectives on self-led CT	• Injunctive and descriptive norm (Citizens’ perceived norm regarding the involvement in self-led CT) • Perceived autonomy (Citizens’ perceived behavioral control)
3. Anticipated impacts of self-led CT on the CT-process	Instrumental and experiential attitude (Citizens’ attitude towards self-led CT)
4. Citizens’ attitude towards the application of self-led CT depends on their own perceived skills and the willingness and skills of others	• Descriptive norm (Citizens’ perceived norm regarding the involvement in self-led CT) • Perceived capacity and perceived autonomy / Self-efficacy (Citizens’ perceived behavioral control)
5. Shame and social stigma may hamper participation in self-led CT	• Experiential attitude (Citizens’ attitude towards self-led CT) • Injunctive and descriptive norm (Citizens’ perceived norm regarding the involvement in self-led CT)
6. Concerns about privacy and data security: a barrier for self-led CT	• Instrumental and experiential attitude (Citizens’ attitude towards self-led CT) • Injunctive norm (Citizens’ perceived norm regarding the involvement in self-led CT) • Perceived capacity and perceived autonomy / Self-efficacy (Citizens’ perceived behavioral control)
7. Citizens’ perspectives and anticipated needs for the implementation and application of self-led CT in practice	• Instrumental and experiential attitude (Citizens’ attitude towards self-led CT) • Injunctive and descriptive norm (Citizens’ perceived norm regarding the involvement in self-led CT) • Perceived capacity and perceived autonomy / Self-efficacy (Citizens’ perceived behavioral control)

*RAA* Reasoned Action Approach, *HBM* Health Belief Model, *CT* Contact tracing

There are, however, a number of limitations that need to be taken into account. Even though we included citizens with a migration background and with varying experiences with CT, most interviewees had a high educational level, were female, and were members of a panel. This does not provide a full representative view of citizens’ perspectives on self-led CT and may have been a potential source for selection bias [53, 54]. We are aware that our sample may be biased towards citizens who are more willing to participate in self-led CT compared to citizens who are more skeptical. However, since the application of self-led CT in practice strongly depends on the willingness of citizens to participate in the first place, understanding the attitudes, perspectives, and needs of relatively willing citizens and fulfilling these in the development and implementation of self-led CT is most crucial. For the individuals that are more skeptical, we may have to find alternative approaches (i.e., fall back on regular CT), or look into ways to get these individuals to participate within a follow-up study. Besides, when interpreting the results, it should be kept in mind that during this phase of the pandemic, the number of cases were increasing, and lockdown measures were (again) in place in the Netherlands. This could have impacted the attitude and motivation of citizens towards self-led CT to a certain extent. For example, citizens may have perceived COVID-19 as less severe compared to the beginning of the pandemic when there was a lot of uncertainty about the severity. This may have led to less need for contact with the PHS for CT, and more of a need for autonomy during the CT-process (i.e., self-led CT). Furthermore, we cannot be sure that what interviewees said during the interviews is also what they would do ‘in real life’ [55]. We are therefore planning a further study, in which we will conduct a pilot study that explores self-led CT in an experimental setting, to gain a better understanding of individuals’ behaviour in this regard. Finally, due to the qualitative nature of our research, we cannot make quantitative statements about the relative importance of certain findings within the current study. As a follow-up study, we, therefore, quantitatively investigated the intention to perform self-led CT including its determinants by distributing online surveys among a larger sample of Dutch citizens (manuscript in preparation).

### Recommendations for practice and future research

Our findings suggest that although regular CT should remain the main method of CT, self-led CT may significantly enhance or support the execution of regular CT. Based on our exploration of citizens’ perspectives, we suggest that for the identification phase, index-cases could be asked to start with the identification of their contact persons after receiving a positive test result (without having to wait for the PHP to contact them). For the contact notification phase, index-cases and PHPs could decide together which contact persons are notified by whom and/or index-cases could notify their contact persons prior to the PHP (via a phone call or using digital tools). For the contact monitoring phase, contact persons could be asked to (digitally) self-report to the PHS when symptoms occur. For index-cases who do not want to, or are not able to participate in self-led CT, the identification and notification of contact persons can be executed as usual in regular CT (i.e., contacted and supported by a PHP). This may help to mitigate the differences in the ability and willingness to participate in self-led CT [56].

Furthermore, the safety of CT-data and privacy is found to be important for both PHPs and citizens, yet the findings highlight a potential trade-off between the individual’s care about privacy and the collection of personal data in CT for the ‘greater good’ (i.e., public health). Using digital tools for CT may, in the context of our study, have impact on the feelings of citizens regarding their privacy since such tools collect (sensitive) health information, which is shared with the PHS. The latter has great value for the PHS since it provides oversight over the CT-process. However, this may conflict with the willingness of citizens to share such information, especially if they are not fully aware of why their own or their contact persons’ personal data are being collected and how these data are going to be used by the PHS. This suggests that such information may be important to address in the implementation of digital tools for self-led CT, since previous studies have shown that to harness the potential of such technologies, they should, amongst other things, be designed around the user’s needs and expectations [42, 57].

Further research is necessary to gain insight into different methods for citizens to identify and notify their contact persons. To this purpose, as mentioned before, we plan to conduct a pilot study among Dutch citizens. In this study we will compare different methods and conditions under which index-cases are willing and able to identify and notify their contact persons via online self-led CT questionnaires.

## Conclusions

Self-led CT may enhance or support the execution of regular CT, and potentially lower the workload for PHPs, through actively involving individuals in tasks that are commonly executed by PHPs (i.e., contact identification, -notification, and -monitoring). Whilst interviewees seem to hold a positive attitude towards self-led CT and using digital tools for this, their intention to make use of self-led CT may depend on prior experiences with regular CT, felt responsibilities and perceived responsibilities of the PHS in CT, anticipated impact of self-led CT on the CT-process, their own perceived skills and the willingness and skills of others, shame and social stigma, and concerns about privacy and data security. During further work on the implementation of self-led CT in practice, the described perspectives and needs of citizens are important to consider, since the success of self-led CT relies heavily on their willingness to make use of this during the CT-process.

### Supplementary Information

**Additional file 1.** Interview guide.

## Data Availability

The data supporting the findings from this study are available from the corresponding author on reasonable request.

## Abbreviations

CT: Contact tracing
PHS: Public health services
PHP: Public health professional
RAA: Reasoned Action Approach
HBM: Health Belief Model
