# Supplementary material for: Citizen involvement in COVID-19 contact tracing with digital tools: a qualitative study to explore citizens’ perspectives and needs
Source: BMC Public Health. 2023 Sep 16;23:1804. doi: 10.1186/s12889-023-16664-x (PMC10504771; doi:10.1186/s12889-023-16664-x)
Supplement: Supplementary file 1 — Additional file 1. Interview guide. [file 12889_2023_16664_MOESM1_ESM.docx]

**Additional file 1. Interview guide**

1. **Introduction**
2. **Background information**

| *Perception and knowledge about CT for COVID-19* | - Are you familiar with the term ‘contact tracing’? - Do you mind if I call it ‘CT’ from now on? - Do you know what CT is? - Could you tell me what you think CT is about/for? - What is your opinion on CT? - What do you think CT is for? |
| --- | --- |
| *Experiences with CT for COVID-19* | - Are you familiar with the term ‘contact tracing’? - Do you mind if I call it ‘CT’ from now on? - What do you think CT is for?   Previously you have been tested positive for the coronavirus and you have been in touch with the PHS for CT. Is that correct?  Previously you have been in contact with someone who had corona and you have been in touch with the PHS for CT. Is that correct?  Try to go back to the moment when you found out you had corona/you had been in contact with someone who had corona.   - Could you tell me what you did at that moment?   Now try to go back to the moment you got in touch with the PHS for CT.   - How did that go? - How did you experience this? What did it bring you? - What did you notice? - Were there aspects that you perceived as unpleasant, or pleasant? Why (not)? - Do you have ideas for the improvement of CT? Why (not)? |
| *Positive (self)test + no CT* | - Are you familiar with the term ‘contact tracing’? - Do you mind if I call it ‘CT’ from now on? - Do you know what CT is? - Could you tell me what you think CT is about/for? - What is your opinion about CT? - What do you think CT is for?   Previously you have been tested positive for the coronavirus.   - Could you tell me what you did at that moment?   Is it correct that you did not get in touch with the PHS for CT? Could you tell me, how did this go?  Previously you have been in contact with someone who had corona.   - Could you tell me what you did at that moment?   Is it correct that you did not get in touch with the PHS for CT? Could you tell me, how did this go? |

1. **Explanation of regular CT via a PowerPoint presentation**

- Is it clear for you what CT consists of? Why (not)?
- What is your opinion on CT?
- Are there certain aspects that you think are unpleasant? Why (not)?
- Are there certain aspects that you think are pleasant? Why (not)?

1. **Explanation of self-led CT via a PowerPoint presentation**
   1. **Contact identification**

| *Attitude* | - How would you feel about this? - What do you think that would be the advantages of doing this? - What do you think that would be the disadvantages of doing this? |
| --- | --- |
| *PBC* | - What would make it easier for you to do this? And more difficult? - What are your reasons to do this (or not)? |
| *Needs* | - What would you need to do this? |

- 1. **Contact notification**

| *Attitude* | - How would you feel about this? - What do you think that would be the benefits of doing this? - What do you think that would be the disadvantages of doing this? |
| --- | --- |
| *PBC* | - What would make it easier for you to do this? And more difficult? - What are your reasons to do this (or not)? |
| *Needs* | - What would you need to do this? |

- 1. **Monitoring of symptoms**

| *Attitude* | - How would you feel about this? - What do you think that would be the benefits of doing this? - What do you think that would be the disadvantages of doing this? |
| --- | --- |
| *PBC* | - What would make it easier for you to do this? And more difficult? - What are your reasons to do this (or not)? |
| *Needs* | - What would you need to do this? |

1. **Explanation of self-led CT with the use of digital tools via a PowerPoint presentation**
   1. **Contact identification**

| *Attitude* | - Is this explanation clear to you? - What is your first impression? - What do you like about it? - What do you dislike about it? |
| --- | --- |
| *PBC* | - Would you make use of such digital tools, via which you can share information about you and your contacts with the PHS to support CT, if you are asked to? - Why (not)? - What would make it difficult for you to identify your contacts in this manner? - What would make it easy for you to identify your contacts in this manner? |
| *Needs* | - What would you need to identify your contacts as accurate as possible? - Would you prefer to do it differently? If yes, how? |
| *Regular CT vs. digital CT* | - Would you see this digital tool as an addition or replacement for the identification of your contacts? Why? - What would be your preferred approach for this? |

- 1. **Contact notification**

| *Attitude* | - Is this explanation clear to you? - What is your first impression? - What do you like about it? - What do you dislike about it? |
| --- | --- |
| *PBC* | - Would you make use of a digital letter to notify your contacts? - Why (not)? - Are there certain contacts you would (or not) notify? Why (not)? - What would make it difficult for you to notify your contacts in this manner? - What would make it easy for you to notify your contacts in this manner? |
| *Needs* | - What would you need to notify your contacts? - Do you have other suggestions for the notification of your contacts? |
| *Regular CT vs. digital CT* | - Would you see this digital tool as an addition or replacement for the notification of your contacts? Why? - What would be your preferred approach for this? |

- 1. **Monitoring of symptoms**

| *Attitude* | - Is this explanation clear to you? - What is your first impression? - What do you like about it? - What do you dislike about it? |
| --- | --- |
| *PBC* | - Would you make use of this tool to monitor your symptoms? - Would you monitor all of your symptoms? - If not: which symptoms would you monitor and which not? - What would make it difficult for you to monitor your symptoms in this manner? - What would make it easy for you to monitor your symptoms in this manner? |
| *Needs* | - What would you need to digitally monitor your symptoms as accurate as possible? |
| *Normal CT vs. digital CT* | - Would you see this digital tool as an addition or replacement for the monitoring of your symptoms? Why? - What would be your preferred approach for this? |

1. **Wrap-up**
